# Efficacy of Prophylactic Dexmedetomidine in Preventing Postoperative Junctional Ectopic Tachycardia After Pediatric Cardiac Surgery

**DOI:** 10.1161/JAHA.116.004780

**Published:** 2017-03-01

**Authors:** Doaa Mohamed El Amrousy, Nagat S. Elshmaa, Mohamed El‐Kashlan, Samir Hassan, Mohamed Elsanosy, Nahed Hablas, Shimaa Elrifaey, Wael El‐Feky

**Affiliations:** ^1^ Pediatric Department Tanta University Hospital Tanta Egypt; ^2^ Cardiothoracic Surgery Department Tanta University Hospital Tanta Egypt; ^3^ Department of Anesthesia & Surgical ICU Faculty of Medicine Tanta University Tanta Egypt

**Keywords:** children, dexmedetomidine, postoperative junctional ectopic tachycardia, Arrhythmias, Cardiopulmonary Resuscitation and Emergency Cardiac Care

## Abstract

**Background:**

Postoperative junctional ectopic tachycardia is one of the most serious arrhythmias that occur after pediatric cardiac surgery, difficult to treat and better to be prevented. Our aim was to assess the efficacy of prophylactic dexmedetomidine in preventing junctional ectopic tachycardia after pediatric cardiac surgery.

**Methods and Results:**

A prospective controlled study was carried out on 90 children who underwent elective cardiac surgery for congenital heart diseases. Patients were randomized into 2 groups. Group I (dexmedetomidine group): 60 patients received dexmedetomidine; Group II (Placebo group): 30 patients received the same amount of normal saline intravenously. The primary outcome was the incidence of postoperative junctional ectopic tachycardia. Secondary outcomes included bradycardia, hypotension, vasoactive inotropic score, ventilation time, pediatric cardiac care unit stay, length of hospital stay, and perioperative mortality. The incidence of junctional ectopic tachycardia was significantly reduced in the dexmedetomidine group (3.3%) compared with the placebo group (16.7%) with *P*<0.005. Heart rate while coming off cardiopulmonary bypass was significantly lower in the dexmedetomidine group (130.6±9) than the placebo group (144±7.1) with *P*<0.001. Mean ventilation time, and mean duration of intensive care unit and hospital stay (days) were significantly shorter in the dexmedetomidine group than the placebo group (*P*<0.001). However, there was no significant difference between the 2 groups as regards mortality, bradycardia, or hypotension (*P*>0.005).

**Conclusion:**

Prophylactic use of dexmedetomidine is associated with significantly decreased incidence of postoperative junctional ectopic tachycardia in children after congenital heart surgery without significant side effects.

## Introduction

Junctional ectopic tachycardia (JET) occurs frequently after pediatric cardiac surgery for congenital heart disease, with an incidence ranging from 2% to 22% and is difficult to manage.^1^ JET is associated with a high incidence of postoperative hemodynamic instability, morbidity, and mortality rate, so preventive measures to decrease its incidence is recommended.^2^ Early postoperative JET usually occurs in the first 48 hours after pediatric cardiac surgery.^3^

Risk factors for the development of postoperative JET in children are variable (eg, younger age, longer cardiopulmonary bypass and aortic cross clamp time, high inotropic score, type of surgery, and electrolyte imbalance.^4^

Many treatment protocols were used to treat postoperative JET, such as the use of digoxin, propranolol, amiodarone, hypothermia, flecainide, and magnesium sulphate.^5^, ^6^, ^7^ Although it is better to prevent JET, none of the current medications are useful in preventing or reducing its incidence after pediatric cardiac surgery except amiodarone, which was studied in our previous study and proved to be effective in preventing JET in children after cardiac surgery.^8^

Dexmedetomidine has been successfully used for sedation and analgesia in pediatric Intensive Care Units (ICUs) after cardiac surgery. It is a selective α‐2 adrenoreceptor agonist that has analgesic, sedative, and anxiety‐decreasing effects. Being selective agonist on α‐2 adrenoceptor limits its action on the central nervous system without activation of α‐1 receptors with unwanted cardiovascular side effects.^9^, ^10^, ^11^, ^12^ The stimulant effect on the α‐2 adrenoceptors decreases the release of catecholamine, producing a sympatholytic action with negative dromotropic and chronotropic effects.^13^, ^14^, ^15^ Dexmedetomidine also depresses both sinus and atrioventricular nodal functions through vagal stimulation.^12^ These sympatholytic actions made the drug useful as a therapeutic option for the prevention of various postoperative tachyarrhythmias. There are few studies on the use of prophylactic dexmedetomidine to decrease the incidence of JET after pediatric cardiac surgery.^9^, ^10^, ^11^

The aim of this study was to assess the efficacy of prophylactic dexmedetomidine in preventing junctional ectopic tachycardia after pediatric cardiac surgery and to study its side effects and impact on various outcomes such as ventilation time, the length of ICU and hospital stay, vasoactive inotropic score, and mortality rate.

## Methods

This is a prospective randomized controlled study conducted at a university hospital and was carried out on 90 children who underwent corrective surgery for congenital heart disease (CHD) between March 2014 and April 2016 after approval by the hospital Ethical Committee and obtaining written informed consents from the parents of studied children.

Inclusion criteria were children aged less than 18 years who underwent cardiac surgery for CHD correction.

Exclusion criteria were known allergy to dexmedetomidine, congestive heart failure, renal impairment, liver diseases, coagulation disorders, permanent pacemaker, history of preoperative arrhythmias, and history of antiarrhythmic medications and β‐blockers within the last 3 days.

Patients were divided into 2 groups:

Group I (dexmedetomidine group): included 60 patients and received dexmedetomidine 0.5 μg/kg diluted in 100 mL of normal saline intravenously over 20 minutes, and the infusion was completed 10 minutes before induction followed by 0.5 μg/kg per hour infusion for 48 hours postoperatively (Precedex; Hospira Worldwide, Lake Forest, IL).
Group II (placebo group): included 30 patients and received the same amount of normal saline intravenously.

The care team and outcome assessors were blinded to the study except the anesthesiologist, who gave the dru g intraoperatively, and he was not a part of the study. Additional bolus doses of midazolam and fentanyl were given when needed. Bispectral index was used to monitor the sedation in the study groups. Data were collected in both the operating room and in the ICU.

All children were premedicated with midazolam 0.1 mg/kg and ketamine 1 mg/kg IV injection. Endotracheal intubation was inserted after giving rocuronium and fentanyl injection. Dexmedetomidine infusions were started as mentioned above. All the surgeries were conducted by the same team of cardiac surgeons and anesthesiologists and with similar techniques during the study period. Inotropes were added when needed.

Inotropic score=(dopamine×1)+(dobutamine×1)+(adrenaline×100)+(noradrenaline×100)+(milrinone×10). Dosages of above drugs were in micrograms per kilogram per minute.^16^

Standard 12‐lead ECG was done in all patients preoperatively and postoperatively at the time of ICU admission. Continuous monitoring of ECG, bispectral index, and oxygen saturation was done with Drager^™^ monitors.

### Diagnostic Criteria for JET

1. Tachycardia with narrow QRS (wide QRS if there was bundle branch block),
2. A ventricular rate more than 170 beats/min,
3. Atrioventricular dissociation with the ventricular rate faster than the atrial rate.

Early‐onset postoperative JET was defined as the occurrence of JET in the first 48 hours postoperatively. Continuous ECG monitoring was used in the ICU. When JET was detected on the monitor, it was also confirmed with a standard ECG strip. The occurrence of bradycardia or hypotension (values ≤5% for age) was also recorded.

Ventilation time was defined as the total time that the patient spent on mechanical ventilation until successful extubation. Duration of ICU and hospital stay after surgery was recorded in days for all patients. Patients who developed postoperative JET were managed by amiodarone, digoxin, and reduction of inotropes.

The primary outcome was the incidence of postoperative JET, hypotension, and bradycardia. Secondary outcomes included vasoactive inotropic score, duration of mechanical ventilation, ICU stay, length of hospital stay, and mortality rate.

### Statistical Analysis

Sample size calculation was calculated before patient recruitment, based on previous studies. A priori power analysis using unequal sized groups with a ratio 2:1 suggested that total sample size of 90 patients achieved a power of 80% (α=0.05) to detect a difference of 23% in the incidence of postoperative JET. We used unequal sized groups because the number of our pediatric patients undergoing corrective surgery for CHD was limited as we are a small cardiothoracic center for CHD and we preferred to increase the number in the dexmedetomidine group for better studying efficacy and side effects of the drug on these vulnerable children.

The Windows version of SPSS 11.0.1 (SPSS Inc., Chicago, IL) was used for statistical analysis. All results are presented in the form of mean±SD. Independent *t* test was used for comparison of continuous variables between the 2 groups. Pearson χ^2^ test was used to compare categorical data. *P*<0.05 was considered significant.

## Results

Preoperative variables (eg, age, sex, weight, preoperative heart rate, and preoperative cyanosis) were recorded and found to be comparable in the 2 groups (*P*>0.05) (Table 1).

**Table 1:** Preoperative Variables

Preoperative Variables	DEX Group (n=60) Mean±SD	Placebo Group (n=30) Mean±SD	*P* Value
Age, months	17.3±4.1	18.3±5.4	0.341^a^
Sex (male:female)	40/20	18 / 12	0.302^b^
Weight, kg	12.4±1.1	12.6±1.7	0.62^a^
Diagnosis			
VSD	16 (26.7%)	10 (33.3%)	0.874^b^
ASD	14 (23.3%)	6 (20%)	
VSD+ASD	8 (13.3%)	5 (16.7%)	
AVSD	10 (16.7%)	5 (16.7%)	
TOF	8 (13.3%)	3 (10%)	
PS	4 (6.7%)	1 (3.3%)	
Preoperative HR	120±7.4	124±7.1	0.077^a^
Cyanosis	12 (20%)	4 (13.3%)	0.122^b^

ASD indicates atrial septal defect; AVSD, atrioventricular septal defect; DEX, dexmedetomidine; HR, heart rate; PS, pulmonary stenosis; TOF, tetralogy of Fallot; VSD, ventricular septal defect.

a Independent *t* test.

b Pearson χ^2^.

There was no significant difference between dexmedetomidine and placebo group as regards cardiopulmonary bypass and aortic cross clamp time. Heart rate while coming off from cardiopulmonary bypass was significantly lower in the dexmedetomidine group compared to the placebo group (*P*<0.001) (Table 2).

**Table 2:** Intraoperative Variables

Intraoperative Variables	DEX Group (n=60) Mean±SD	Placebo Group (n=30) Mean±SD	*P* Value
ACC time, minutes	94.5±12.8	98.6±9.6	0.12
CPB time, minutes	130.4±12.1	132.3±9.8	0.46
HR while coming off CPB	130.6±9	144±7.1	<0.001

ACC indicates aortic cross clamp; CPB time, cardiopulmonary bypass; DEX, dexmedetomidine; HR, heart rate.

The incidence of postoperative JET was significantly lower in the dexmedetomidine group (*P*=0.02). It was 3.3% (2 out of 60) in the dexmedetomidine group, while it was 16.7% (5 out of 30) in the placebo group. The 2 cases of JET in dexmedetomidine group were as follows: 1 case of tetralogy of Fallot (TOF) and 1 case of pulmonary stenosis,. while in the placebo group there were 5 cases with JET, 2 tetralogy of Fallot, 1 pulmonary stenosis, 1 ventricular septal defect (VSD), and 1 atrioventricular septal defect. Vasoactive inotropic score was significantly lower in the dexmedetomidine group than the placebo group (*P*<0.001) (Table 3).

**Table 3:** Postoperative Variables

Postoperative Variables	DEX Group (n=60) Mean±SD	Placebo Group (n=30) Mean±SD	*P* Value
JET incidence (%)	2 (3.3%)	5 (16.7%)	0.02^a^
VIS, μg/kg per minute	11.3±2.5	13.5±2.2	<0.001^b^
VT, hours	12.7±4.9	21.6±6.1	<0.001^b^
PCCU stay, days	1.8±0.7	4.2±1.2	<0.001^b^
Length of hospital stay, days	5.8±0.7	9.3±1.9	<0.001^b^
Mortality rate (%)	1 (1.7%)	2 (6.7%)	0.217^a^
Bradycardia (%)	1 (1.7%)	1 (3.3%)	0.368^a^
Hypotension (%)	1 (1.7%)	1 (3.3%)	0.618^a^

DEX indicates dexmedetomidine; JET, junctional ectopic tachycardia; PCCU, Pediatric Cardiac Care Unit; VIS, vasoactive inotropic score; VT, ventilation time.

a Pearson χ^2^.

b Independent *t* test.

Mean ventilation time, and duration of ICU and hospital stay (in days) were significantly reduced in the dexmedetomidine group compared to the placebo group (*P*<0.001). There was 1 mortality in the dexmedetomidine group, while there were 2 mortalities in the placebo group in the postoperative period, but this was found to be of no statistical significance (*P*=0.475). There was no significant difference between the 2 groups as regards the occurrence of bradycardia and hypotension (Table 3). All cases of hypotension and bradycardia were mild, not necessitating treatment or stoppage of dexmedetomidine.

## Discussion

JET is one of the most serious and life‐threatening postoperative arrhythmias that is difficult to manage. JET can cause serious hemodynamic deterioration that is poorly tolerated after pediatric cardiac surgery. To the best of our knowledge, the present study is the first prospective study to assess the efficacy of intraoperative prophylactic dexmedetomidine in preventing JET after variable congenital heart surgery in pediatrics.

The major finding of our study is that prophylactic dexmedetomidine decreased the incidence of postoperative JET in pediatric patients undergoing cardiac surgery from 16.7% in the placebo group to 3.3% in the dexmedetomidine group, which was in agreement with the results of previous studies.^17^, ^18^ The incidence of postoperative JET in our study was lower than the 2 previous studies because they performed their research only on patients with tetralogy of Fallot, which is known to have a high frequency of postoperative JET. On the other side, Shuplock et al results showed that postoperative administration of dexmedetomidine was not associated with a clinically significant difference in the incidence of postoperative tachyarrhythmia after CHD surgical correction in children.^19^ This could be explained by their administration of dexmedetomidine postoperatively, while in our study we started administration of dexmedetomidine intraoperatively just before induction of anesthesia.

Also, our study found that heart rate while coming off from cardiopulmonary bypass was significantly lower in the dexmedetomidine group, and this decreased the incidence of postoperative JET. These results are in keeping with the results of Hammer et al,^20^ who found that dexmedetomidine depressed the function of both sinus and atrioventricular nodes, decreasing heart rate and consequently reducing the incidence of postoperative JET. Also, Kamibayashi et al^21^ found that epinephrine/halothane–induced ventricular tachycardia can be prevented by dexmedetomidine through activation of α2‐adrenoreceptors.

The main mechanism of action of dexmedetomidine, by decreasing heart rate and consequently decreasing the incidence of postoperative JET, is through its negative chronotropic effect. Dexmedetomidine leads to activation of central presynaptic α_2A_ adrenergic receptors, in turn leading to decreasing the release of norepinephrine.^22^ Meanwhile, stimulation of α_2A_ adrenergic receptors in the dorsal motor nucleus of the vagus nerve consequently increases vagal output to the heart.^23^ Thus, the decrease in heart rate can be considered a combination of decreased sympathetic outflow from the central nervous system and increased vagal discharge to the myocardium. However, other studies suggested another mechanism of action through activation of cerebral imidazoline receptors.^20^

Sedatives and analgesics are cornerstones in the postoperative management of the ventilated pediatric cardiac patient. They have many adverse effects such as respiratory depression, bradycardia, hypotension, tolerance, dependence, and so on. Dexmedetomidine has recently been used in many ICUs because of its good sedative and analgesic effects without respiratory depression.^13^, ^14^, ^15^ Its main side effect is bradycardia; however, this is used for prevention and control of different tachyarrhythmias such as JET after cardiac surgery.

Most cases of JET occur in tetralogy of Fallot patients, pulmonary stenosis, and VSD. This could be attributable to increased risk of direct trauma to conduction system, or infiltrative hemorrhage to the bundle of His by resection or excision of muscle bundles as in the case of repair of tetralogy of Fallot or pulmonary stenosis or edema from sutures close to the conduction system as in VSD repair.^24^

The current study showed that inotropic score was significantly lower in the dexmedetomidine group compared to the placebo group. The dexmedetomidine group required less inotropes, which can be explained by better hemodynamics because of decreased incidence of JET. This was in agreement with the findings of Kadam et al.^17^

We found that dexmedetomidine significantly shortened ventilation time, and postoperative ICU and hospital stay. This can be explained by better hemodynamics in the dexmedetomidine group with less JET incidence and better pain control. This was achieved without significant side effects, mainly bradycardia and hypotension.

Limitations of the study: The study involved a small number of patients. Diagnosis of JET depends on the monitor and strip ECG because atrial wire study was unavailable.

## Conclusion

Prophylactic use of dexmedetomidine is associated with a significantly decreased incidence of postoperative JET in children after congenital heart surgery without significant side effects.

## Disclosures

None.
