# Expression of Concern

**DOI:** 10.1161/JAHA.119.014585

**Published:** 2020-08-14

The article "Efficacy of Prophylactic Dexmedetomidine in Preventing Postoperative Junctional Ectopic Tachycardia After Pediatric Cardiac Surgery," by El Amrousy et al, which published on March 1, 2017, and appeared in the March 15, 2017 issue of the journal, (*J Am Heart Assoc*. 2017;6:e004780. DOI: 10.1161/JAHA.116.004780) contains substantial overlap with a previously published article by El‐Shmaa et al, “The efficacy of pre‑emptive dexmedetomidine versus amiodarone in preventing postoperative junctional ectopic tachycardia in pediatric cardiac surgery,” which published online in *Annals of Cardiac Anaesthesia* on October 7, 2016 (*Ann Card Anaesth*. 2016 Oct‐Dec;19(4):614‐620).

Specifically, certain elements in the demographics and results presented in Tables 1‐3 in both articles appear to be nearly identical. These included the sex ratio and preoperative heart rate of the placebo/control groups in Table 1 of each article; the intraoperative heart rate of the placebo/control groups in Table 2 of each article; and the Vasoactive Inotropic Score, Ventilation Time, the Pediatric Cardiac Care Unit Length of Stay, the Hospital Length of Stay, and incidence of mortality/bradycardia/hypotension in Table 3 of each article.

We are communicating with the authors involved in these publications. We are publishing this Expression of Concern while we await the outcome of these communications and to indicate that the data and statements in the listed publications may not be reliable.

